# Methotrexate‐associated proliferative disorder in the lower esophagus extending to the gastroesophageal junction: A case report

**DOI:** 10.1002/deo2.14

**Published:** 2021-08-15

**Authors:** Yuki Hojo, Masafumi Takatsuna, Satoshi Ikarashi, Hiroteru Kamimura, Rika Kimura, Masaki Mito, Yusuke Watanabe, Yusuke Tani, Junji Yokoyama, Shuji Terai

**Affiliations:** ^1^ Division of Gastroenterology and Hepatology, Graduate School of Medical and Dental Sciences Niigata University Niigata Japan; ^2^ Division of Molecular and Diagnostic Pathology, Graduate School of Medical and Dental Sciences Niigata University Niigata Japan

**Keywords:** case report, gastroesophageal junction (EGJ), methotrexate (MTX), methotrexate‐associated lymphoproliferative disorder (MTX‐LPD), rheumatoid arthritis (RA)

## Abstract

A 64‐year‐old woman was receiving oral methotrexate (MTX) for rheumatoid arthritis (RA) for 15 years. She underwent esophagogastroduodenoscopy because of discomfort in the chest. Endoscopic findings revealed an ulcer in the lower esophagus extending to the gastroesophageal junction (EGJ). The ulcer occupied half of the esophageal lumen and had a sharp and clear margin. Magnifying narrow‐band imaging endoscopy revealed the deposition of white plaque, and there were few microvessels in the edge and bottom of the ulcer. Histologic examination of the biopsy specimens from the oral edge of the lesion revealed proliferation of atypical lymphoid cells (immunophenotype results: CD20 [+], CD3 [partially +], CD5 [−], and BCL‐2 [−]]. The patient was diagnosed with methotrexate‐associated lymphoproliferative disorder (MTX‐LPD) and was advised to stop MTX intake. After 2 months of stopping MTX, the ulcer was found to be almost regressed and showed signs of healing. MTX‐LPD in the lower esophagus extending to the EGJ is extremely rare. This case can help in expanding the understanding of esophageal MTX‐LPD.

## INTRODUCTION

Methotrexate (MTX) is a highly effective drug used for the treatment of rheumatoid arthritis (RA). The occurrence of methotrexate‐associated lymphoproliferative disorder (MTX‐LPD) has been reported.^1^ There are several reports of MTX‐LPD in the body of the stomach and duodenum^2^, ^3^, ^4^, ^5^; however, there are no reports of MTX‐LPD occurring in the lower part of the esophagus up to the gastroesophageal junction (EGJ). We report a case of MTX‐LPD in the EGJ in a patient with RA who was on oral MTX treatment.

## CASE REPORT

**FIGURE 1:**
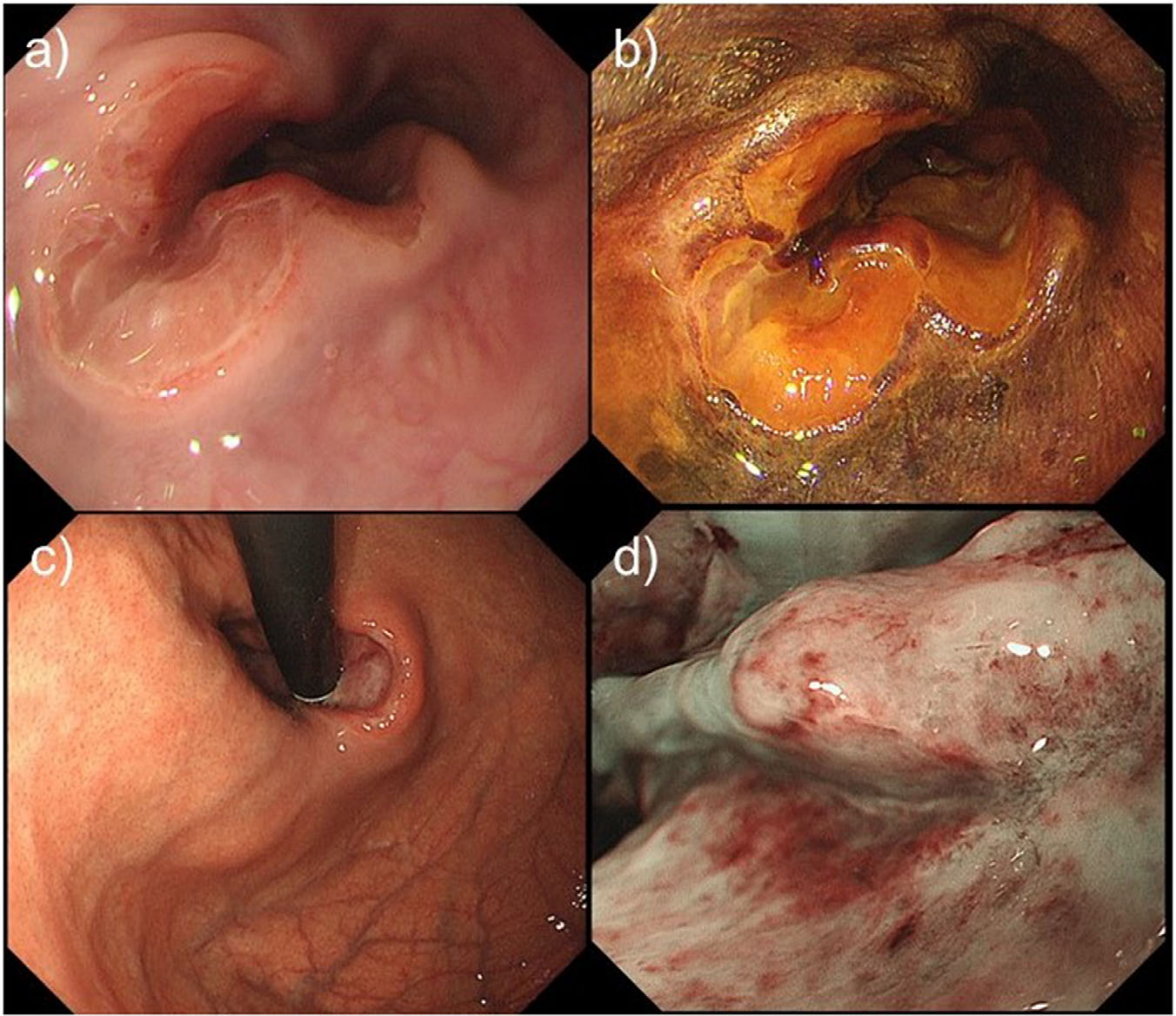
(a) Esophagogastroduodenoscopy findings reveal an ulcer on the lower part of the esophagus. (b) Lugol chromoendoscopy reveals that the ulcer has a voiding lesion. (c) Endoscopic findings from the gastroesophageal junction side reveal that the ulcer has a sharp margin. (d) Magnifying narrow‐band imaging endoscopy finding.

**FIGURE 2:**
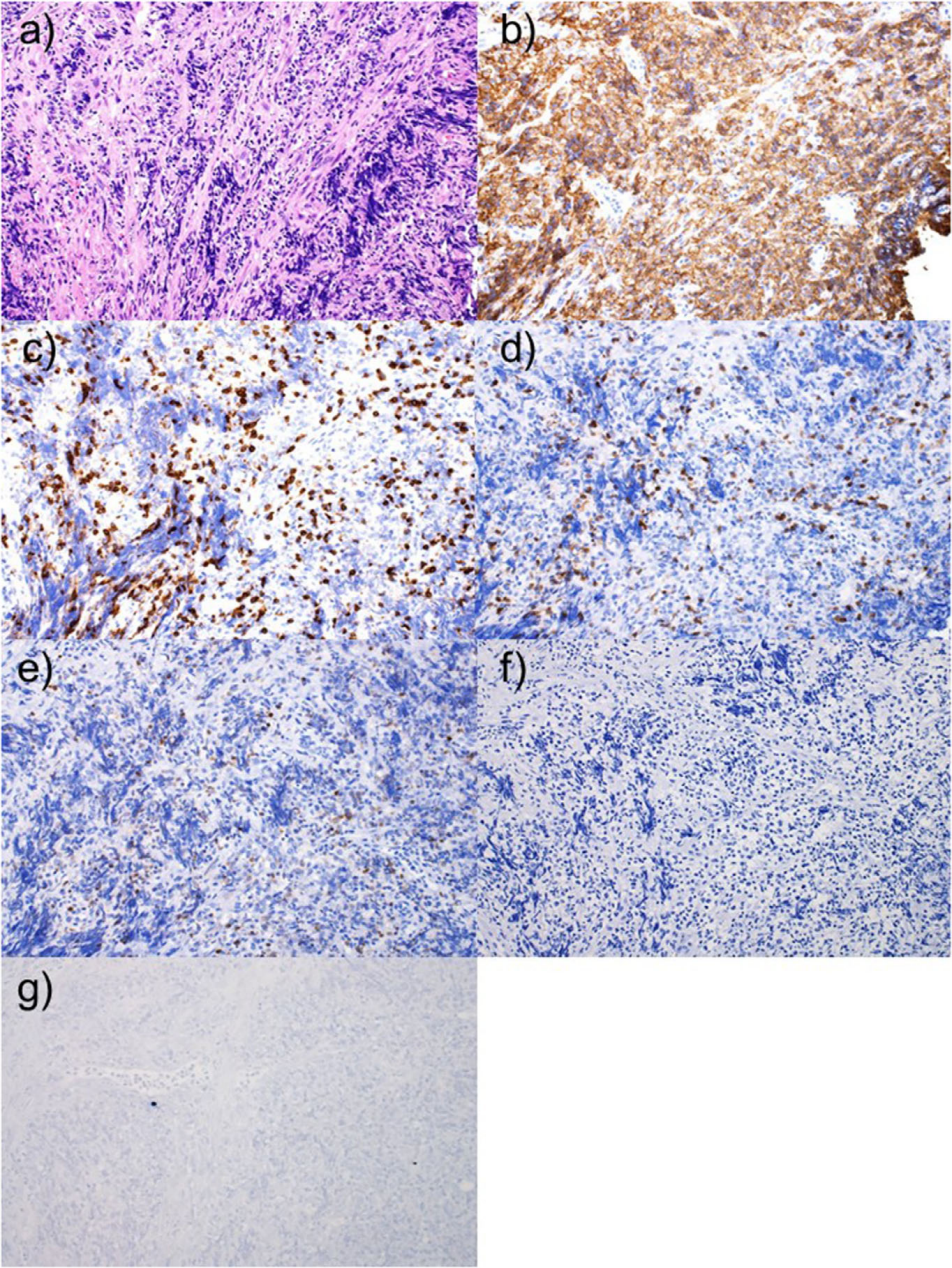
Histologic examination of biopsy specimens from the ulcer edge. (a) Hematoxylin–eosin staining reveals diffuse proliferation of the atypical lymphoid cells (200×). (b) Immunohistochemical staining for CD20 is positive in the atypical lymphocytes (200×). (c) Immunohistochemical staining for CD3 is partially positive in the atypical lymphocytes (200×). (d) Immunohistochemical staining for CD5 is negative (200×). (e) Immunohistochemical staining for BCL‐2 is negative (200×). (f) Immunohistochemical staining for cytokeratin AE1/AE3 is negative (200×). (g) Epstein–Barr virus‐encoded RNA in situ hybridization shows negative staining (200×).

**FIGURE 3:**
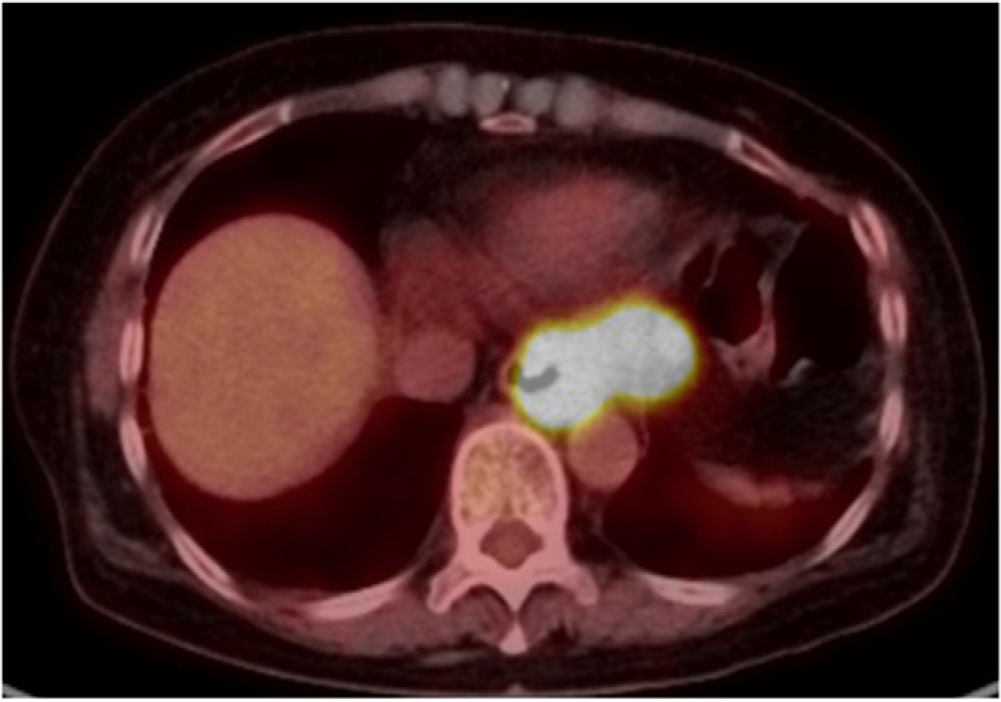
Positive emission tomography–computed tomography scan shows focal uptake from the lower esophagus to the gastroesophageal junction.

**FIGURE 4:**
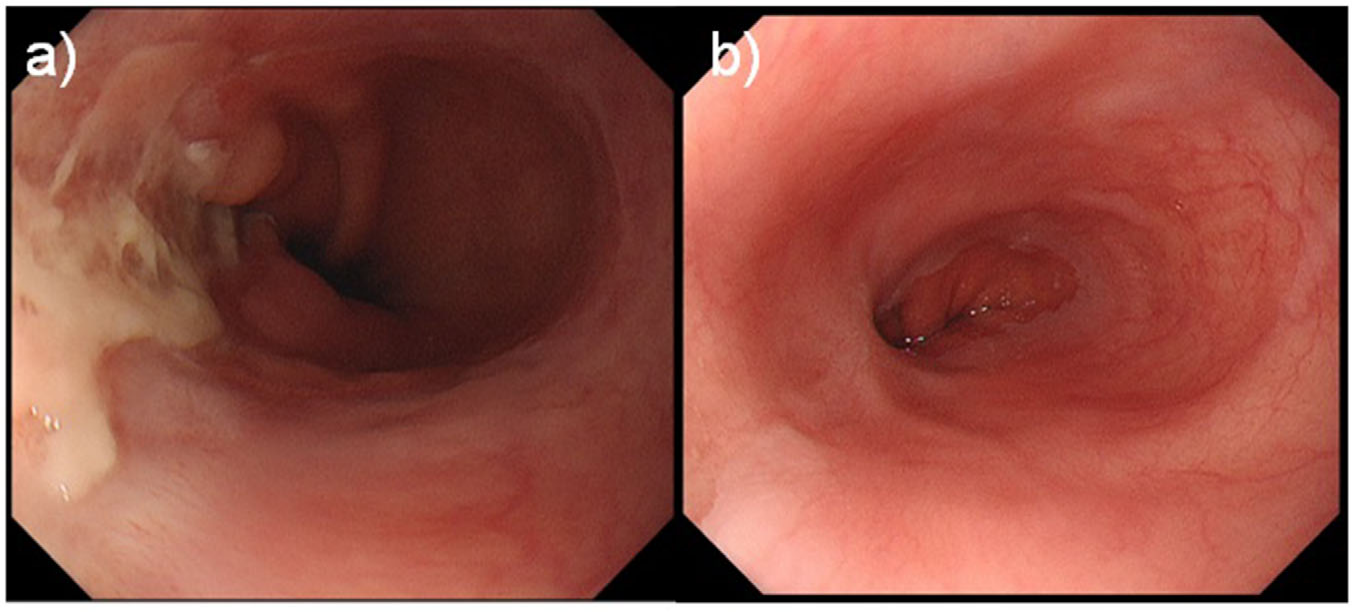
(a) After 2 months of stopping methotrexate, the ulcer is found to be almost regressed and shows signs of healing. (b) After 6 months, the ulcer is healing more.

A 64‐year‐old woman with RA was on oral MTX treatment for 15 years, and was also taking prednisolone for the same. She underwent esophagogastroduodenoscopy because of discomfort in the chest. There was no history of fever, chills, or night sweats. Endoscopic findings revealed an ulcer on the lower esophagus (35 cm from the incisor teeth) extending to the EGJ (Figure 1a). The ulcer occupied half of the esophageal lumen and had a sharp and clear margin. Lugol chromoendoscopy revealed that the ulcer had a voiding lesion (Figure 1b). Endoscopic findings from the gastric side revealed that the ulcer margin was raised resembling a submucosal tumor (SMT) (Figure 1c). Magnifying narrow‐band imaging (M‐NBI) endoscopy revealed the deposition of white plaque, and there were few microvessels in the edge and bottom of the ulcer (Figure 1d). Histologic examination of biopsy specimens from the ulcer edge revealed diffuse proliferation of the atypical lymphoid cells (Figure 2a). Immunohistochemical staining for CD20 was positive in the atypical lymphocytes (Figure 2b). Immunohistochemical staining for CD3 was partially positive in the atypical lymphocytes (Figure 2c). Immunohistochemical staining for CD5, BCL‐2, and cytokeratin AE1/AE3 was negative in the atypical lymphocytes (Figure 2d–f). Epstein–Barr virus‐encoded RNA in situ hybridization showed negative staining (Figure 2g). Positron emission tomography–computed tomography scan showed only focal uptake from the lower esophagus to the EGJ; however, there was no significant uptake throughout the chest, mediastinal lymph nodes, abdomen, or pelvis (Figure 3). The ulcer was diagnosed as malignant lymphoma‐associated B‐cell type. A hematologist was consulted, who diagnosed the case as MTX‐LPD. MTX withdrawal was suggested. After 2 months of withdrawal of MTX, the ulcer almost regressed, and a part of the ulcer showed signs of healing (Figure 4a). After 6 months of MTX withdrawal, the ulcer healed significantly (Figure 4b), and the condition of the patient improved. She was prescribed a proton pump inhibitor for heartburn and acid reflux, and was followed‐up.

This study was conducted in accordance with the principles outlined in the Declaration of Helsinki and was approved by the ethics review board of Niigata University (no. 2192). Additionally, informed consent was obtained from the patient.

## DISCUSSION

Ellman et al.^6^ reported in 1991 that MTX‐LPD leads to lymphoma in patients with RA on MTX therapy. The World Health Organization guideline has classified MTX‐LPDs as “other iatrogenic immunodeficiency‐associated LPDs.”^7^ This disease is more common in patients aged 60–70 years, and these patients usually have a history of receiving MTX for ≥5 years.^1^, ^8^ Discontinuation of MTX improves the condition in 30%–50% of the cases; however, if there is no improvement or deterioration in the condition, treatment of malignant lymphoma is warranted.^1^ In the present case, the patient was on MTX therapy for >5 years, and the condition improved after MTX withdrawal. If no remission is achieved even after the withdrawal of MTX, additional chemotherapy such as R‐CHOP (rituximab, cyclophosphamide, doxorubicin, vincristine, prednisolone) might be required.^1^

Cases of MTX‐LPD of the gastrointestinal tract have been reported; however, most of the lesions were localized in the stomach. No case of MTX‐LPD in the esophagus extending to the EGJ has been reported. One of the possible etiologies of MTX‐LPD is the dysregulation of the immune system due to Epstein–Barr virus infection.^8^ In the stomach, the lesions of MTX‐LPD are mostly confined in the body without involving the EGJ. In the present case, MTX‐LPD was found from the lower esophagus to the EGJ; however, the mechanism is unclear. In the present case, the patient showed a negative Epstein–Barr virus infection result. Therefore, we hypothesized that the interaction of both inflammation due to gastroesophageal reflux and immunity imbalance due to MTX caused the proliferation of atypical lymphocytes, which struck MTX‐LPD, because she complained of heartburn and acid reflux after MTX withdrawal. Additionally, we did not evaluate the possibility of CMV and HSV. This evaluation might have provided further understanding about the pathophysiology of LPD on both the esophagus and EGJ. Endoscopic findings of LPD, including malignant lymphoma of the intestinal tract, are also diverse, including ulceration, SMT‐like ridges, and polypoid.^9^, ^10^ The margins of these ulcers are clearly demarcated, and an SMT‐like ridge is observed.^2^, ^3^, ^4^, ^5^ MTX‐LPD extending from the EGJ to the lower esophagus also has a similar feature. M‐NBI findings of MTX‐LPD in the stomach have shown abnormal blood vessels.^2^ In the present report, few microvessels in the edge and bottom of the ulcer were found. This might be attributed to the proliferation of atypical lymphocytes along with the rapid damage of normal tissue.

To conclude, MTX‐LPD in the lower esophagus extending to the EGJ is extremely rare. This case can help in expanding the understanding of esophageal MTX‐LPD.

## CONFLICT OF INTEREST

The authors declare no conflict of interest.

## FUNDING INFORMATION

None.
